# Evaluation of cytotoxic effects and acute and chronic toxicity of aqueous extract of the seeds of *Calycotome villosa* (Poiret) Link (subsp. *intermedia*) in rodents

**Published:** 2018

**Authors:** Badiaa Lyoussi, Khadija Cherkaoui Tangi, Nicole Morel, Mohamed Haddad, Joelle Quetin-Leclercq

**Affiliations:** 1 *Laboratory of Physiology-Pharmacology and Environmental Health, University Sidi Mohamed Ben Abdallah, Fez, Morocco*; 2 *Institute of Neuroscience, Université catholique de Louvain, Brussels, Belgium*; 3 *GNOS research group, Louvain Drug Research Inistitute,* *Université catholique de Louvain, Brussels, Belgium*

**Keywords:** Calycotome villosa (Link subsp. intermedia) seed, Traditional medicine, Cytotoxicity, Acute and chronic toxicity, Clinical Chemistry, Hematology

## Abstract

**Objective:**

The present investigation was carried out to evaluate the safety of an aqueous extract of the seeds of *Calycotome villosa *(Poiret) Link (subsp. *intermedia)* by determining its cytotoxicity and potential toxicity after acute and sub-chronic administration in rodents.

**Materials and Methods:**

Cytotoxic activity was tested in cancer and non-cancer cell lines HeLa, Mel-5, HL-60 and 3T3. Acute toxicity tests were carried out in mice by a single oral administration of *Calycotome* seed-extract (0 - 12 g/kg) as well as intraperitoneal doses of 0 - 5 g/kg. Sub-chronic studies were conducted in Wistar rats by administration of oral daily doses for up to 90 days. Changes in body and vital organ weights, mortality, haematology, clinical biochemistry and histologic morphology were evaluated.

**Results:**

The lyophilized aqueous extract of *C. villosa* exhibited a low cytotoxicity in all cell lines tested with an IC_50 _> 100 µg/ml. In the acute study in mice, intra-peritoneal administration caused dose-dependent adverse effects and mortality with an LD_50_ of 4.06 ± 0.01 g/kg. In the chronic tests, neither mortality nor visible signs of lethality was seen in rats. Even AST and ALT were not affected while a significant decrease in serum glucose levels, at 300 and 600 mg/kg was detected. Histopathological examination of the kidney and liver did not show any alteration or inflammation at the end of treatment.

**Conclusion:**

In conclusion, the aqueous extract of *C. villosa* seed appeared to be non-toxic and did not produce mortality or clinically significant changes in the haematological and biochemical parameters in rats.

## Introduction

Herbal remedies are commonly employed in developing countries to treat various diseases. 

This practice is an alternative to compensate for some perceived deficiencies in orthodox pharmacotherapy (Aniagu et al., 2005 ▶). There is limited scientific evidence regarding the safety and efficacy of these herbal remedies to support their continued therapeutic application and their utilization is often based on long-term clinical experience. For these reasons, a systematic toxicological evaluation of plants and their diverse active components are being conducted, to examine the credibility of their traditional uses (Zhu et al., 2002 ▶). 

*Calycotome villosa *(Poiret) Link subsp. *intermedia *belongs to the Papilionacea family. It is an erect shrub that can grow up to 2 m tall, especially in the north of Africa and Spain (Greuter et al., 1989 ▶). The flowers are yellow and they appear during spring; their seeds are ripen in June. This plant is used for various ethnomedical purposes in Morocco. Evaluation of this plant is based on its traditional use by a substantial population in the middle north of Morocco. It is used as an infusion and administered for varying lengths of time to treat rheumatism and a variety of diseases, such as wound scar (Bellakhdar J. 1997 ▶). Chrysin glucoside, an active component originating from the plant, has been demonstrated to have diuretic, vasodilator and hypotensive effects in rats (Cherkaoui et al., 2008 ▶). 

The phytochemical studies of *Calycotome villosa* subsp. *intermedia *have led to the isolation and structural elucidation of alkaloids from the seeds (El Antri et al., 2004a ▶, El Antri et al., 2004b ▶) and flavonoid glycosides from the flowers and leaves (El Antri et al., 2004c ▶). 

To the best of our knowledge, there is no record in the literature on the toxicity profile of *C. villosa*. Acute and sub-chronic toxicity data may be required to predict the safety and effects of long-term exposure to a particular medicinal plant.

Our study was therefore undertaken to determine the cytotoxicity, as well as the acute and the sub-chronic toxicity profiles of *C. villosa* seeds.

## Materials and Methods

**Plant material**

The seeds of *C. villosa *subsp*. intermedia* were collected in Zrireg valley, plateau of Tazzeka, area of Taza, Morocco and stored at room temperature in a dark, dry place prior to use. Authentic samples were identified at the Department of Biology, Faculty of Science, Sidi Mohamed Ben Abdellah University Fès, Morocco, where a voucher specimen was deposited (reference number LB134).

**Preparation of the extracts**

The seeds of *C. villosa* subsp. *intermedia* were washed with water, dried in an oven at 40 °C and then powdered in a Willey mill. The lyophilised aqueous extract was prepared by adding 500 mL of distilled water to 50 g of the powder followed by heating the mixture under reflux for 20 min. Next, the boiled decoction was centrifuged, filtered, frozen at −20 °C and then lyophilised (FreeZone® Dry 4.5, USA). The crude yield of the extracted material was approximately 9 % w/w; it was stored at -20 ^o^C in the dark until further use.

**Cytotoxicity study of** ***C. villosa*** **seed-extract in cancer and non-cancer cell lines** ***in vitro***

HL-60 cells were cultured with Flow RPMI 1640 medium supplemented with 10 % heat-inactivated foetal-bovine serum, 0.33 % L-glutamine, 1 % non-essential amino acids, 1% sodium pyruvate and penicillin-streptomycin (100 IU/mL-100 µg/mL). Mouse 3T3 fibroblasts, human HeLa and melanoma Mel-5 cell lines were grown in Gibco MEM supplemented with 10 % heat-inactivated foetal-bovine serum and penicillin (100 IU/mL). Cells were incubated in a humidified atmosphere with 5 % CO_2_ at 37 °C as previously described (Stévigny C et al., 2002 ▶). Stock solution of *C. villosa* seed-extract was prepared at 10 mg/ml in distilled water. The effects of the extract were evaluated using tetrazolium salts MTT and WST-1 colorimetric assays based on the cleavage of the reagent by mitochondrial dehydrogenases in viable cells (Mosmann, 1983 ▶). 50,000 HL-60 cells in 100 µL medium, 5000 HeLa and Mel-5 cells and 10,000 3T3 cells in 200 µL medium were seeded in each well of 96 well-plates.

Then, 100 µL of fresh medium containing various concentrations of *C. villosa* seed-extract or vehicle were added to HL-60 wells. Other cells were incubated for 24 hr, then, the medium was removed and replaced by 200 µl/well fresh medium containing various concentrations of *C. villosa* seed-extract or vehicle at the same final concentration. Each concentration was tested in at least 6 wells. After 72 hr treatment, the medium was supplemented with 10 µL of WST-1 for HL-60. The medium of the other cell lines was removed and replaced by 100 µL of DMEM (without serum) containing 10 µL of MTT (3 mg/ml in PBS). After 45 min incubation, the medium containing MTT was removed, and 100 µL of DMSOwas added to each well. In the two cases, plates were shacked and absorbance recorded at two wavelengths (450-620 nm for WST-1 and 570-620 nm for MTT) against a background control as blank. The relative absorbance was expressed as the percentage of the corresponding control considered as 100 %. Campthothecin was used as positive cytotoxic reference compound (Huang Rl *et al.,* 1998). The results are expressed as IC_50_ values (concentration of extract causing 50 % inhibition of cell growth).

**Acute toxicity study of** ***C. villosa*** **seed-extract in mice**

Healthy adult mice (IOPS OFA) of either sex were obtained from the animal colony of our department (original strain procured from Iffa-Credo, l’Arbresle, France), weighing average 24 g were divided into 8 groups of 12 (6 males and 6 females).

The animals were acclimatized in cages under standard environmental conditions of light/dark cycles (12 h/12 h), temperature (23 ± 1 °C), and frequent air changes. Animals had free access to tap water and standard pellet diet, except for a short fasting period of 2 h before the treatment with the single doses of the lyophilised *C. villosa* Cseed-extract.

The lyophilised extract was diluted with distilled water (1 g/mL) on the day of the experiment, and administered either orally or intraperitoneally. The doses given were increased progressively so that each dose was 100 % higher than the previous one (Kennedy et al., 1986 ▶), while the control group received the vehicle only. The general behaviour change and signs of toxicity and mortality were observed continuously for 1 hr after the treatment, and then intermittently for 4 hr and thereafter over a period of 24 hr (Twaij et al., 1983 ▶). The mice were further observed once a day for up to 14 days following treatment for behavioural changes and signs of toxicity and/or death, as well as the latency of death (Silva et al., 2007 ▶). The LD_50_ values were determined according to the method of Litchfield and Wilcoxon (1949).

**Sub-chronic toxicity of** ***C. villosa*** **seeds extract in rats**

Adults male Wistar rats (250-300 g) were randomly divided into four groups of six animals each. The animals were separated by gender and housed three in each cage under the same conditions as mentioned above for the mice. 

The first group served as a control and was given equivalent volumes of water while the remaining three groups were orally administered with 150, 300, and 600 mg/kg BW of lyophilised aqueous extract of *C. villosa *seeds, for 90 days. The lyophilised aqueous extract was diluted in distilled water (1 g/mL). Toxic manifestations and mortality were monitored daily. At the end of each 30-day period, body weights were recorded and blood was collected from the retro-orbital puncture (Waynforth, 1980 ▶) in two tubes with or without anticoagulant (ethylenediamine tetraacetate). Blood collected in tubes without the anticoagulant agent was allowed to clot before centrifugation (4000 rpm at 4 °C for 10 min) to obtain serum, which was stored at −20 °C until biochemical analysis, while the anticoagulated blood was analysed immediately for haematological parameters.

The care and handling of the animals were in accordance with the internationally accepted standard guidelines for use of animals, and the protocol was approved by our institutional committee on animal care following the French Technical Specifications for the Production, Care and Use of the Laboratory Animals.

**Measurement of biochemical and haematological parameters in rats**

Serum glucose (Neese et al., 1976 ▶), creatinine, urea (Tabacco et al., 1979 ▶), alanine aminotransferase (ALT (SGPT)) (IFCC, 1980 ▶), aspartate aminotransferase (AST (SGOT)) (IFCC, 1975 ▶), and total bilirubin were determined enzymatically using specific kits by measurement of the optical density of the reaction products at the corresponding wavelength using a spectrophotometer (BioSystems BTS-310 photometer). The haematological parameters including total red cell (RBC), leukocyte (WBC), lymphocytes, neutrophils, platelets, haematocrit, and haemoglobin were determined using automatic analyser (Sysmex KX-21).

**Histopathological studies of the liver and kidney tissues**

At the end of the 90-day treatment, rats were anesthetized by diethyl ether and sacrificed by decapitation. Then, the kidney and liver were excised from each rat. The isolated tissues were washed with normal saline and immersion fixed in 10 % buffered formalin immediately upon removal. They were gradually dehydrated, embedded in paraffin, cut into 5 µM sections and stained with haematoxylin and eosin for histological examination according to the standard procedure (Ross et al., 1989 ▶).

Different organs (heart, spleen, liver, lungs and kidneys) were carefully disserted and their absolute weights were determined. The relative organ weight of each animal was then calculated as follows:

$\mathrm{Relative organ weight}=\frac{\mathrm{Absolute organ weight}(g)}{\mathrm{Body weight of rat on the day animals were sacrificed}(g)}\times 100$

**Drugs**

All chemicals were obtained from Sigma chemicals except for WST-1 which was obtained from Boehringer. The kits for biochemical analyses were obtained from Biosysteme (Morocco).

**Statistical analysis**

Results were expressed as mean ± S.E.M. The difference among experimental and control groups were determined using the statistical software Graph Pad Prism Version 4.0. Significance difference was determined by Student’s *t-*test and analysis of variance (ANOVA) followed by Bonferroni test. p-values less than 0.05 were considered significant.

## Results

**Cytotoxicity study of** ***C. villosa*** **seed-extract on cancer and non-cancer cell lines** ***in vitro***

 The lyophilised aqueous extract of *C. villosa* seeds was tested for their ability to inhibit the growth of HeLa, Mel-5, and HL-60 cancer cell and 3T3 non cancer-cells, *in vitro*.

As seen in Table 1, treatment of several cell lines with the seeds extracts resulted in a relatively high IC_50_ value (higher than 100 µg/ml).

**Table 1:** Cytotoxic effect of lyophilised aqueous extract of *C. villosa* seeds. Camptothecin was used as positive control.

**IC**_50 _ **(µg/mL)** ^a^				
**Compound**	HL-60**	Mel-5*	HeLa *	3T3 *
***C. villosa *** **seeds**	99± 23	108±17	103±13	120±45
**Camptothecin**	0.0053 µM	0.45 µM	0.038 µM	2.8 µM

a IC_50_ :Concentration of the extract causing 50% inhibition of mitochondrial activity.

* MTT test and

** WST-1 test.

**Table 2a:** Effect of single oral doses of lyophilised aqueous extract of *C. villosa* seeds in mice.

**Dose (g/kg)**	**Sex**	**D/T**	**Latency **	**Symptoms**
**0**	M F	0/6 0/6	- -	None None
**1**	M F	0/6 0/6	- -	None None
**2**	M F	0/6 0/6	- -	None None
**4**	M F	0/6 0/6	- -	None None
**6**	M F	0/6 0/6	- -	None None
**8**	M F	0/6 0/6	- -	None None
**10**	M F	0/6 1/6	- >48hr, <60hr	None Hypoactivity
**12**	M F	1/6 2/6	>48hr, <60hr >48hr, <60hr	Hypoactivity, piloerection Hypoactivity, piloerection, trembling, breathing difficulty

Acute toxicity study – following administration of oral doses of 0-12 g/kg. The lyophilised aqueous extract of *C. villosa* seeds, dissolved in distilled water, was administered orally. All treated mice (n=12 in each group; 6 males and 6 females) were carefully examined for up to 14 days for adverse effects causing behavioural changes, lethality and latency of death. D/T=No. of dead mice/No. of treated mice. None = no symptoms observed during the observation period; latency= lag time to death following administration of *C. villosa* extract.

**Acute toxicity study of** ***C. villosa*** **seeds extract in mice**

The no-observed-adverse-effect level (NOAEL) of *C. villosa *seed-extract was 8 g/kg while the lowest-observed-adverse-effect level (LOAEL) was 10 g/kg (Alexeeff et al., 2002 ▶) when given via the oral route (Table 2a). Similarly, for the intraperitoneal route, NOAEL of *C. villosa* seed-extract was 2.5 g/kg while LOAEL was 3 g/kg (Table 2b). The mortality rate as well as the acute toxicity of the orally and the intraperitoneally administered *C. villosa *seed- extract increased progressively as the dose increased from 10 g/kg to 12 g/kg and from 3 g/kg to 5 g/kg, respectively (Tables 2a & 2b).

**Table 2b:** Effect of single intraperitoneal doses of lyophilised aqueous extract of *C. villosa* seeds in mice.

**Dose (g/kg)**	**Sex**	**D/T**	**Latency **	**Symptoms**
**0**	M F	0/6 0/6	- -	None None
**1.0**	M F	0/6 0/6	- -	None None
**1.5**	M F	0/6 0/6	- -	None None
**2.0**	M F	0/6 0/6	- -	None None
**2.5**	M F	0/6 0/6	- -	None None
**3.0**	M F	0/6 1/6	- >36hr, <60hr	None Hypoactivity
**3.5**	M F	1/6 2/6	>36hr, <60hr >24hr, <48hr	Hypoactivity Hypoactivity, trembling
**4.0**	M F	2/6 3/6	>36hr, <48hr >24hr, <36hr	Asthenia, trembling, breathing difficulty, polyuria Asthenia, trembling, breathing difficulty, polyuria
**4.5**	M F	3/6 5/6	>24hr, <36hr >24hr, <36hr	Asthenia, trembling, breathing difficulty, polyuria Asthenia, trembling, breathing difficulty, polyuria
**5.0**	M F	6/6 6/6	>24hr, <36hr >24hr, <36hr	Asthenia, trembling, breathing difficulty, polyuria Asthenia, trembling, breathing difficulty, polyuria

Acute toxicity of intraperitoneally administered doses (0-5 g/kg). The lyophilised aqueous extract of *C. villosa* seeds, dissolved in distilled water, was administered by the intraperitoneal route. All treated mice (n=12 in each group; 6 males, 6 females) were carefully examined for up to 14 days for adverse effects causing behavioural changes, lethality and latency of death. D/T=No. of dead mice/No. of treated mice. None=no symptoms observed during the observation period; latency= lag time to death following administration of *C. villosa* extract.

In this study, the main behavioural signs of toxicity were atypical locomotion, asthenia, trembling, piloerection, and urination. Asthenia, hypoactivity and urination were noticed immediately after oral and intraperitoneal administration of plant extract and were more pronounced at the higher doses and persisted until death. There appeared to be a gender difference in terms of the acute toxicity of *C. villosa* seeds extract (adverse effects, mortality and death latency) given by the oral as well as the intraperitoneal route (Tables 1a & 2b), the females being more sensitive than the males.

**Figure 1:**
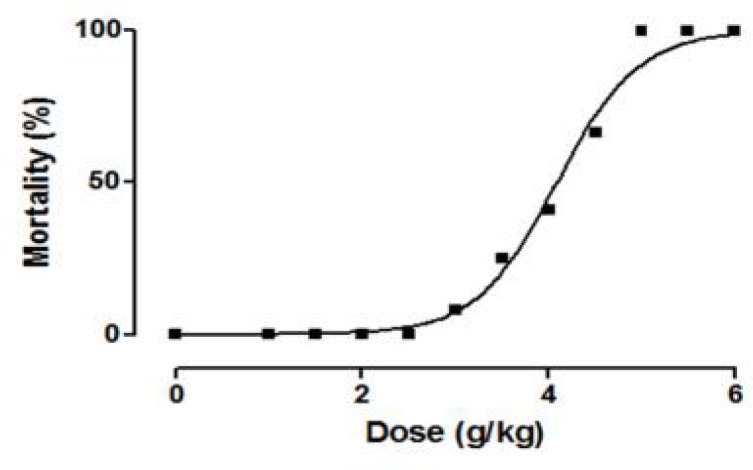
Dose-mortality curve for lyophilised aqueous extract of *C. villosa* seeds in mice (administered with a single intraperitoneal dose). LD_50_ = 4.06 ± 0.01 g/kg.

The acute oral and intraperitoneal toxicity (LD_50_) of *C. villosa *seeds extract in mice were calculated to be 10.32 ± 0.01 g/kg and 4.06 ± 0.01g/kg, respectively (Figure 1). The LD_50_ was lower for female mice (3.87 g/kg) than for male mice (4.24 g/kg) after intraperitoneal administration; however, no significant difference was detected after oral administration. 

**Sub-chronic toxicity of** ***C. villosa*** **seeds extract in rats**

*Clinical observations and body weight of rats*

Daily oral administration of *C. villosa *seeds extract to rats for 90 days, did not produce any obvious symptoms of toxicity or mortality up to the highest dose tested (600 mg/kg). However, as presented in Table 3, rats treated with the *C. villosa *seeds extract (300 or 600 mg/kg) showed a significant reduction (p<0.01) in weight gain as compared to control rats receiving water, while weight gain in rats treated with 150 mg/kg of the extract appeared to be suppressed. On the 90th day of the study, compared to the baseline value, the control rats gained 15% weight, while rats treated with *C. villosa* seeds extract at the dose of 150 mg/kg, gained only 2.7%. However, rats treated with 300 and 600 mg/kg of *C. villosa *seeds extract did lose weight by4.5 % and7.7 %, respectively.

There were no significant differences (p>0.05) in the weights of the liver, heart, kidneys and other organs expressed as percent of body weight among the control and the treated groups at the end of the experiment (Table 4).

*Biochemical and haematological parameters of rats*

**Figure 2:**
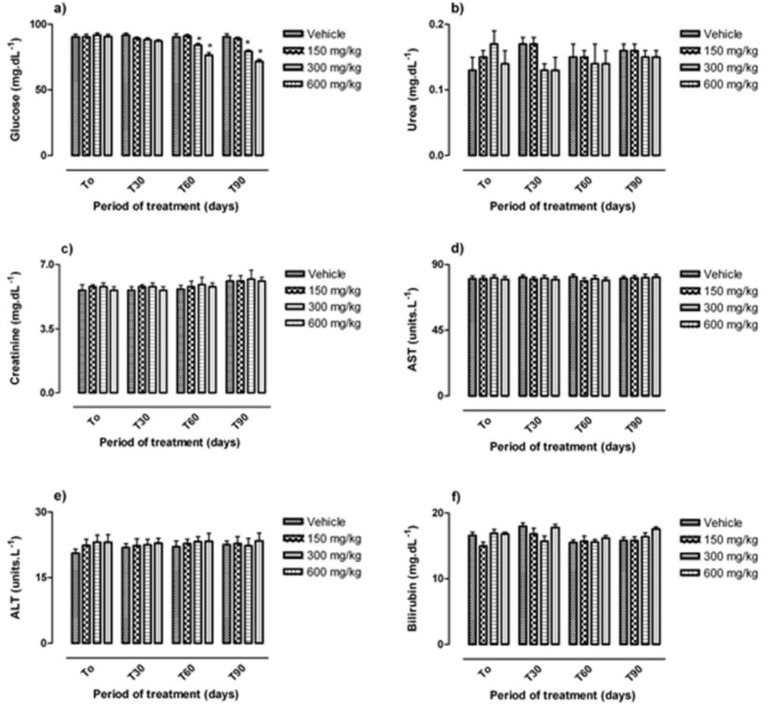
Effect of chronic oral administration of lyophilised aqueous extract of *C. villosa* seeds on biochemical parameters in rats. The lyophilised aqueous extract of the plant was given daily by the oral route to groups of Wistar rats at the following doses: 0 mg/kg (Vehicle), 150 mg/kg, 300 mg/kg, and 600 mg/kg for up to 90 days. Biochemical parameters were measured before treatment (T_0_); 30 days (T_30_); 60 days (T_60_) and after 90 days of treatment (T_90_).

The effect of sub-chronic daily oral administration of *C. villosa *seeds extract on biochemical parameters is presented in Figure 2. Serum glucose levels of rats treated with *C. villosa *seeds extract (300 and 600 mg/kg) were significantly decreased (p<0.001) as compared to the controls (Figure 2a and Table 5).Furthermore,, we can observe significant dose and time effect. while no significant effect of 150 mg/kg of *C. villosa *seeds extract on glucose levels was observed up to the 90th day of treatment.

**Figure 3:**
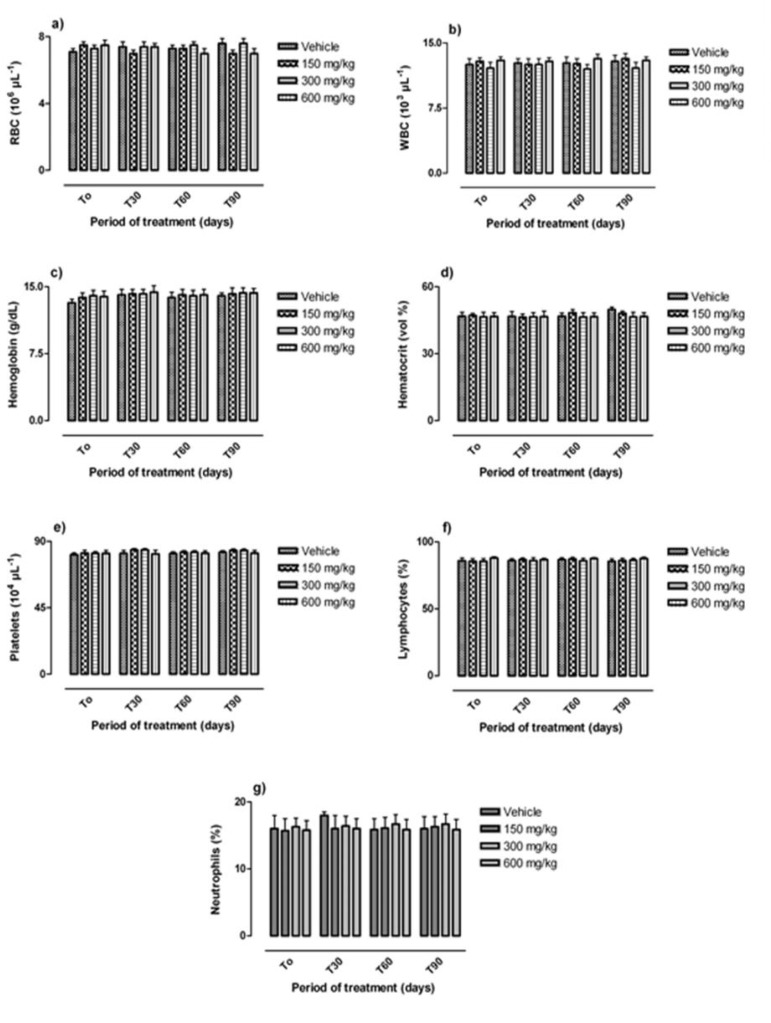
Effect of chronic oral administration of lyophilised aqueous extract of *C. villosa *seeds on haematological parameters in rats. The lyophilised aqueous extract of the plant was given daily by the oral route to groups of Wistar rats at the following doses: 0 mg/kg (Vehicle), 150 mg/kg, 300 mg/kg, and 600 mg/kg for up to 90 days. Haematological parameters were measured before treatment (T_0_); 30 days (T_30_); 60 days (T_60_) and after 90 days of treatment (T_90_). Panel (a): Red blood cell (RBC); Panel (b): White blood cell (WBC); Panel (c): Hemoglobin; Panel (d): hematocrit; Panel (e): Lymphocyte; Panel (f): Platelets; Panel (g): Neutrophils. Data are expressed as mean   SEM.

No significant changes in serum creatinine, urea, bilirubin and the activity of liver enzymes (ALT and AST) in rats treated with all three doses of *C. villosa *seeds extract daily for 90 days were observed (p<0.05) (Figures 2b, c, d, e and f). Daily oral administration of *C. villosa* seeds extract had no significant effect on hematological parameters (neutrophils, lymphocytes, platelets, haemoglobin, hematocrit, red blood cells, and total leukocytes) of treated and control rats. The results are summarized in Figure 3

**Table 3:** Body weight of rats treated with lyophilised aqueous extract of *C. villosa* seeds (oral doses of 0-600 g/kg BW for up to 90 days) as a function of the duration of treatment.

	**% of change in body weight (g)**			
	**Treatment** ** (mg/kg BW)**			
**Treatment Period (Days)**	**Control**	**150**	**300**	**600**
**T**_0_	257 ± 3	259 ± 3	262 ± 3	260 ± 3
**T**_30_	275 ± 4*** (+7%)	262 ± 2 (+1.15%)	258 ± 2 (-1.5%)	253 ± 3** (-2.7%)
**T**_60_	290 ± 4*** (+12.8)	264 ± 3 (+1.9%)	254 ± 2 ** (-3.05%)	245 ± 3 ** (-5.77%)
**T**_90_	296±3 *** (+15.1%)	266 ± 4 (+2.7%)	250 ± 2 ** (-4.5%)	240 ± 3 ** (-7.7%)

Values are means ± S.E.M for n=6.

( ):% change in weight loss or gained.

*** p<0.001 compared to values at T_0._

** p<0.01 compared to values at T_0_.

**Table 4:** Relative organ weights of rats at the end of treatment with lyophilised aqueous extract of *C. villosa *seeds (oral doses of 0-600 g/kg BW for up to 90 days).

	**Relative organ weight (g%) body weight**			
	**Treatment (mg/kg BW)**			
**Organ**	**Control**	**150**	**300**	**600**
Liver	3.59 ± 0.42	3.22 ± 0.55	3.65 ± 0.46	3.80 ± 0.28
Heart	0.41±0.06	0.42±0.05	0.42 ±0.16	0.42 ± 0.03
Lungs	0.77 ± 0.17	0.79 ±0.22	0.62 ± 0.21	0.65 ± 0.62
Spleen	0.49 ± 0.59	0.43 ± 0.89	0.40 ± 0.08	0.46 ± 0.12
Kidneys	3.59 ± 0.42	3.33 ±0.36	3.65 ± 0.46	3.80 ± 0.28

Values are means ± S.E.M for n=6.

Mean data on the same row carrying the same superscript do not differ significantly from each other (p>0.05).

**Table 5:** Hypoglycemic effect of daily oral dosing of lyophilised aqueous extract of *C. villosa *seeds in rats; expressed as % of change in serum glucose levels; oral doses of 0-600 g/kg BW for up to 90 days.

	**% of change in serum glucose levels (mg/dL)**			
	**Treatment (mg/kg BW)**			
**Treatment Period (Days)**	**Control**	**150**	**300**	**600**
**T**_0_	90.4±2	90.5±1.9	91.6±1.9	90.8±1.6
**T**_30_	+1.43%	-0.54%	-3.71%	-5.06%
**T**_60_	+0.7%	+0.5%	-8.29%**	-15.84% ***
**T**_90_	+1.21%	-1.54%	-13.42%***	-21.14% ***

Values are means ± S.E.M for n=6.

*** p<0.001 compared to values at T_0_.

** p<0.01 compared to values at T_0_

Panel (a): Glucose; Panel (b): Urea; Panel (c): Creatinine; Panel (d): Aspartate aminotransferase (AST); Panel (e): Alanine aminotransferase (ALT); Panel (f): Bilurubin.

Data are expressed as mean S.E.M for n=6; (*) p0.05 versus the values at T_0_.

*Histopathogical analysis*

*Kidney morphology*

Histological examination of sections of the kidneys from rat treated with *C. villosa *seeds extract (150-600 mg/kg) showed no marked microscopic changes compared to control.

**Figure 4:**
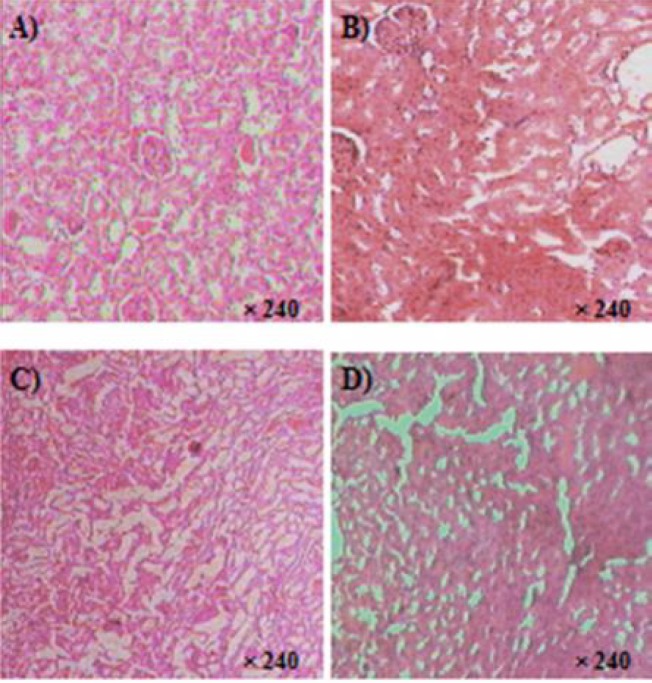
Photomicrographs of the sections of the kidney showing normal features in rats treated orally with lyophilised aqueous extract of *C. villosa *seeds at the following doses: A) 0 mg/kg (Vehicle), B) 150 mg/kg, C) 300 mg/kg, and D) 600 mg/kg for up to 90 days of for up to 90 days. Histopathological examination was carried at the end of treatment. HES: X 240.

As shown in Figure 4, no cortical tubular vacuolations or interstitial mononuclear cell infiltration was observed.

*Liver morphology*

**Figure 5:**
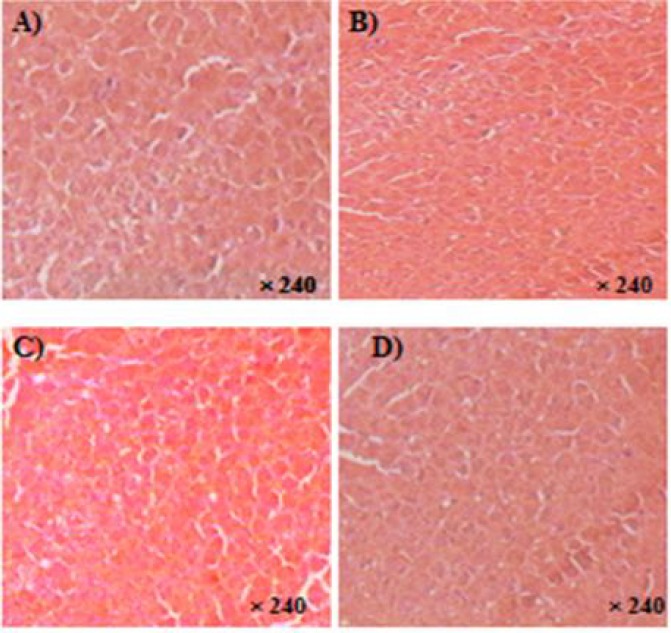
Photomicrographs of the sections of the liver showing normal features in rats treated orally with lyophilised aqueous extract of *C. villosa *seeds at the following doses: A) 0 mg/kg (Vehicle), B) 150 mg/kg, C) 300 mg/kg, and D) 600 mg/kg for up to 90 days of for up to 90 days. Histopathological examination was carried at the end of treatment. HES: X240

Livers of rats treated with *C. villosa *seeds extract (150-600 mg/kg) appeared normal, both macroscopically and microscopically. Histopathological examination of liver from treated and control animals showed normal hepatic lobules, central venule, and liver cells of normal shape and size. No signs of congestion, inflammation, cellular necrosis or cholestasis were seen in liver sections of the four groups (Figure 5).

## Discussion

In the present study, we showed that the lyophilised aqueous extract of *C. villosa *subsp*. intermedia *seeds, used in the traditional medicine for a number of diseases in Morocco, was relatively non–toxic based on the scale proposed by our study.

The effect of *C. villosa* seeds extracts on cell growth of HeLa, Mel-5, HL-60 and 3T3 was investigated in this study. As shown in Table 2, more than 50 % of cells were viable at 100 µg/mL (IC_50_> 100 µg/ml). The obtained IC_50_ is relatively high for *in vitro* tests. It seems that the extract exhibit relatively low cytotoxic effects. However, in order to confirm that this extract is not cytotoxic/low cytotoxicity, additional tests are needed, such as the LDH leakage assay.

To our knowledge, this is the first report on cytotoxic evaluations of this subspecies of *C. villosa *(subsp*. intermedia*)*. *The essential oil of *C. villosa *(Poiret) Link leaves and the methanol extract in toto were reported previously to possess cytotoxicity against Vero cell line at 0.04µl/ml(Loy et al., 2001 ▶). So *C. villosa *(subsp*. intermedia*) seeds extract is less cytotoxic, but further studies are required to verify the absence of cytotoxicity of isolated molecules from this subspecies (El Antri et al., 2004a ▶; El Antri et al., 2004b ▶; El Antri et al., 2004c ▶) to determine the safety of drugs and plant products for human traditional use.

The acute toxicological evaluation of *C. villosa *seeds extract in mice (IOPS OFA) revealed an oral LD_50_= 10.32 g/kg. The extract did not exhibit any signs of adverse effects (NOAEL) within the 14 days of treatment. As reported by (Grossel and Crowl, 1994 ▶), an LD_50_ between 5 and 15 g/kg determined after single oral doses in rats, is considered practically non-toxic in humans. Moreover, it has also been noted that mice are more susceptible to oral toxicity compared to humans (VanMiert, 1989 ▶). 

To determine the inherent toxicity of *C. villosa *seeds extract, it was administered via intraperitoneal route to mice (IOPS OFA) and the LD_50 _was calculated to be 4.06 g/kg (Figure 1). This route of administration was not likely to be used in humans; but, it gave us more details and a guideline for selecting doses for the chronic low-dose study, which may be more clinically relevant. 

The adverse effects of drugs and toxic substances are also more common in women than in men (Ebert et al., 1998 ▶; Drici et al., 2001 ▶; Liechti et al., 2001 ▶); so, we reported also a gender difference in acute toxicity of *C. villosa *seeds extracts. This difference has been reported by others regarding some plant extract such as *Alstonia scholaris* (Baliga et al., 2004 ▶), *Artemisia afra* (Mukinda and Syce, 2007 ▶) and *Cylicodiscus gabunensis* (Kouitcheu Mabeku et al., 2007 ▶).

In this study, we also demonstrated that sub-chronic oral administration of *C. villosa *seeds extracts to rats did not show any signs of hepatotoxicity nor nephrotoxicity (Normal levels of bilirubin and urea throughout the study) as assessed by biochemical measurements and by histopathological examination. No effect was observed, except a significant decrease in body weight and plasma glucose levels in rats treated with *C. villosa *seeds extract at the highest concentrations (300 and 600 mg/kg BW), as compared to the control group. 

Increased or decreased body weight has been used as an indicator of adverse effects of drugs and chemicals (Raza et al., 2002 ▶; Teo et al., 2002 ▶). The reduction of body weight gain in this study may be a result of decreased appetite and thereby lower caloric intake by the animals, as has been shown for certain plants and their constituents, such as ephedrine and active compounds in the genus *Ephedra* (Avula et al., 2006 ▶; Gazda et al., 2006 ▶; Rebecca et al., 2002 ▶), saponin from Korean red ginseng (Kim et al., 2005 ▶), aristocholic acids isolated from several medicinal plants (Lee et al., 2002 ▶), and galegine from *Verbesina encelioides* (Lopez et al., 1996 ▶).Nevertheless, the aqueous extract of *C. villosa *seeds did not produce any demonstrable toxic effect based on the relative weights of the liver, spleen, testes, and kidneys at all doses tested.

A significant decrease (21 %) in plasma glucose levels in normoglycemic rats revealed the hypoglycemic activity of the *C. villosa *seeds extract at the highest doses used, and was in concordance with the reduction of body weight observed in this study. Further experiments are required in order to establish the hypoglycemic activity of *C. villosa *seeds extract in experimental diabetic animals and determine if it affects the absorption of glucose and sugars, or the appetite, which could both account for the decreased weight. 

Certain medicinal herbal preparations or conventional drugs or chemicals adversely affect various blood components (King et al., 1984 ▶). Decrease or increase in blood cell counts and depletion of plasma constituents or their elevation beyond acceptable reference range could equally demonstrate hematoxicity (Dioka et al., 2002 ▶; Olson et al., 2000 ▶). 

The *C. villosa *seeds extract did not affect the hematograms of the rats in a manner that would suggest adverse effects on their bone marrow, which is a source of recticulocytes. Some herbal remedies may also have hepato- and nephro- toxic effects (Akdogan et al., 2003 ▶; Lin et al., 2003 ▶). Damage to these organs often results in the elevation of clinical chemistry parameters such as some serum enzymes - aspartate aminotransferase (AST) and alanine aminotransferase (ALT), as well as total and conjugated bilirubin, urea, and creatinine (Kallner et al., 1989 ▶). Our results show that *C. villosa *seeds extract did not induce significant changes in the histology of the liver and kidneys. In addition, the bilirubin results indicatethat the hepatic capacity to excrete bilirubin was not impaired by *C. villosa *seeds extract.

Taken together, our results suggest that the lyophilised aqueous extract of *C. villosa *subsp*. intermedia *seeds could be considered as not cytotoxic, and did not cause any signs of mortality or organ toxicity in both acute and chronic toxicity studies in rodents except at high doses in acute tests; therefore, it can considered as relatively safe for consummation in the traditional medicine. The decreases in both plasma glucose levels and body weight need further investigation, and may suggest that the seeds of this plant could be useful as an appetite suppressant in the treatment of obesity. More additional clinical toxicological evaluations (organ toxicity, and risk-benefit analysis) should be performed to define a safe dose and protect the population from possible toxic effects of the plant.
